# Bidirectional relationship between premenstrual disorders and autoimmune disease: a nationwide register-based study in Sweden

**DOI:** 10.1192/j.eurpsy.2025.367

**Published:** 2025-08-26

**Authors:** J. Zhou, E. Bränn, Y. Yang, Y. Chen, M. Opatowski, U. A. Valdimarsdóttir, E. Bertone-Johnson, D. Lu

**Affiliations:** 1 Institute of Environmental Medicine, Karolinska Institutet, Stockholm, Sweden; 2 Faculty of Medicine, University of Iceland, Reykjavik, Iceland; 3 Department of Biostatistics and Epidemiology, School of Public Health and Health Sciences, University of Massachusetts Amherst, Amherst, United States

## Abstract

**Introduction:**

Prementrual disorders (PMD) affect millions of women in reproductive age worldwide. Understanding the potential link between PMD and its comorbidities, including autoimmune disease (AD), is crucial for ultimately improving women’s health. Although hormonal fluctuations seem involved in the development of PMD and some AD, the relationship between PMD and AD remains unclear.

**Objectives:**

Hence, we aimed to investigate the bidirectional association between PMD and AD.

**Methods:**

Leveraging Swedish nationwide and regional registers, we conducted a nested-case control study and a matched-cohort study. Among 3,630,028 eligible women of reproductive ages during 2001-2022, we identified 104,972 incident PMD, their unaffected full sisters, and 10 unaffected matched women per case. We extracted 41 types of AD diagnosis recorded in the registers. Using conditional logistic regression and Cox regression models, we estimated the odds ratio (OR) and hazard ratio (HR) with 95% confidence interval (CI).

**Results:**

Women with AD had a 12% increased risk of subsequent PMD (95% CI 1.10-1.15) compared to unaffected women, and a 6% increased risk compared to their unaffected full sisters (95% CI 1.01-1.11). Women with PMD had 23% (95% CI 1.20-1.26) and 17% (95% CI 1.10-1.24) elevated risks of subsequent AD compared to unaffected women and full sisters, respectively. Among parous women, a stronger bidirectional association was observed for those both exposed to PMD and perinatal depression (nested case-control OR:1.46, 95%CI 1.38-1.54; matched cohort HR:1.49, 95%CI 1.34-1.66) compared to only PMD (nested case-control OR:1.04, 95%CI 1.00-1.07; matched-cohort HR:1.14, 95%CI 1.07-1.21). The strongest bidirectional association with PMD was found for autoimmune thyroid disease and celiac disease.

**Conclusions:**

Our findings illustrate a bidirectional association between PMD and AD, in particular among parous women with a history of perinatal depression. If confirmed in future studies, healthcare professionals need to be vigilant about the risk of AD in women with PMD and *vice versa.*

**Disclosure of Interest:**

None Declared

